# Adverse pregnancy outcomes in hypertensive disorders of pregnancy: an in-depth comparison between subtypes

**DOI:** 10.3389/fmed.2026.1817901

**Published:** 2026-06-08

**Authors:** Xu Zhou, Yinglan Wu, Ting Han, Xiaoying Chen

**Affiliations:** NHC Key Laboratory of Birth Defect for Research and Prevention (Hunan Provincial Maternal and Child Health Care Hospital), Changsha, Hunan, China

**Keywords:** adverse pregnancy outcomes, gestational hypertension, hypertensive disorders of pregnancy, preeclampsia-eclampsia, subtypes

## Abstract

**Objective:**

To compare adverse pregnancy outcomes (APOs) between subtypes of hypertensive disorders of pregnancy (HDP).

**Methods:**

Data were obtained from the Maternal Near-Miss Surveillance System in Hunan Province, China, 2012–2022. HDP was classified into four subtypes: preeclampsia-eclampsia (PE), chronic hypertension (CH), chronic hypertension with superimposed preeclampsia (CHSP), and gestational hypertension (GH). Pearson’s chi-square tests were used to determine if there were significant differences in the prevalence of APOs between HDP subtypes.

**Results:**

A total of 780,359 pregnant women were included, and 38,397 HDPs were identified, including 17,782 PEs (46.31%), 15,889 GHs (41.38%), 3,340 CHs (8.70%), and 1,386 CHSPs (3.61%). The overall prevalence of APOs among pregnant women with HDP was as follows: anemia (30.78%), diabetes mellitus (20.33%), preterm birth (19.53%), low birthweight (18.74%), hemorrhage disorder (9.07%), infection (4.71%), liver disease (2.55%), stillbirth and neonatal death (2.38%), maternal near-miss (1.57%), kidney disease (0.98%), heart disease (0.65%), pulmonary disease (0.11%), embolism (0.03%), and maternal death (0.02%). There were significant differences in the prevalence of all APOs between HDP subtypes, except for pulmonary disease, embolism, and maternal death (*p* < 0.05). Compared with other subtypes, anemia was more common in PE (32.97%), diabetes mellitus was more common in CH (25.27%) and CHSP (25.11%), preterm birth was more common in CHSP (37.01%) and PE (27.76%), low birthweight was more common in CHSP (31.39%) and PE (27.72%), hemorrhage disorder was more common in PE (9.86%), infection was more common in PE (5.20%) and CHSP (5.05%) than other subtypes; liver disease was more common in CHSP (4.11%), stillbirth and neonatal death were more common in CHSP (6.64%) and PE (3.18%), maternal near-miss was more common in PE (2.47%) and CHSP (2.31%), kidney disease was more common in CHSP (3.97%) and CH (1.44%), heart disease was more common in CHSP (1.15%) and CH (0.96%).

**Conclusion:**

Most APOs were more common in both CHSP and PE than other subtypes, including preterm birth, low birthweight, infection, stillbirth and neonatal death, and maternal near-miss; diabetes mellitus, liver disease, kidney disease, and heart disease were common in CHSP but not PE; anemia and hemorrhage disorder were common in PE but not CHSP; diabetes mellitus, kidney disease and heart disease were common in CH. Our finding is useful for clinical counseling and future in-depth studies.

## 1 Introduction

Hypertensive disorders of pregnancy (HDP) are a group of maternal disorders characterized by rising blood pressure during pregnancy, with systolic blood pressure > = 140 mm Hg and/or diastolic blood pressure > = 90 mm Hg (1). The global observed prevalence of HDP was 5.2–8.2% (2). The prevalence of HDP was 7.30% in China (a meta-analysis in 2021) (3), and 7.39% in Hunan Province, China, in 2022 (4). In summary, it is a common complication of pregnancy.

HDP is usually classified into four subtypes: preeclampsia-eclampsia (PE), chronic hypertension (CH), chronic hypertension with superimposed preeclampsia (CHSP), and gestational hypertension (GH) (3, 5). There were significant differences in the prevalence of subtypes. The prevalence of PE, GH, CH, and CHSP in China were 4.50, 3.30, 0.60, and 0.60%, respectively (3).

Not only is the prevalence of HDP high, but it also constitutes a serious risk to both pregnant women and their offspring. HDP was the second leading cause of maternal death (6); Even in developed countries, HDP (especially preeclampsia) is one of the leading causes of maternal mortality (7). It is reported that HDP increases the risk of perinatal death by fivefold (7). In addition, HDP is associated with a series of adverse pregnancy outcomes (APOs), such as maternal near-miss, preterm birth, low birth weight, stillbirth and neonatal death, et al. (4, 8). Furthermore, HDP is associated with future chronic hypertension (9, 10), and cardiovascular disease in pregnant women and their offspring (11–13). In summary, HDP poses serious health risks.

Clinical experience shows that there are significant differences in the clinical symptoms of different subtypes of HDP. Previous studies also showed that there were differences in the relationship between HDP subtypes and APOs (14–16). However, to the best of our knowledge, there relatively fewer studies have compared APOs between HDP subtypes, including China. Most of the existing studies have focused on several APOs such as preterm birth and low birthweight, while a large number of other complications are less frequently addressed. In addition, fewer studies have been conducted using long-term, large-scale, large-sample surveillance data.

Therefore, in this study, we aimed to compare various APOs across HDP subtypes using long-term, large-area, and large-sample surveillance data from Hunan Province, China, from 2012 to 2022. Hunan Province is located in south-central China and covers a population of about 65 million. Our study may significantly contribute to the field.

## 2 Methods

### 2.1 Data sources

This study used data from the Maternal Near-Miss Surveillance System in Hunan Province, China, 2012–2022, run by the Hunan Provincial Health Commission, and involved 18 representative registered hospitals in Hunan Province. These 18 hospitals are well-distributed throughout the province and are well-represented. In each of the 18 hospitals sampled, data were collected for all pregnant or postpartum women admitted to obstetrics departments.

Surveillance data of all pregnant and postpartum women, including HDP and APOs, were collected using a specially designed data collection form. This surveillance method is used throughout China, and the information collection methods and indicator definitions were consistent with the WHO standards (17, 18). For further details on data collection methods, please refer to the National Maternal Near Miss Surveillance Working Manual (18).

### 2.2 Indicators and definitions

The definition and diagnosis of HDP (including subtypes) complied with the 2021 International Society for the Study of Hypertension in Pregnancy classification, diagnosis, and management recommendations for international practice (1). Hypertension is defined as systolic BP ≥ 140 and/or diastolic BP ≥ 90 mm Hg. HDP is usually classified into four subtypes (3, 5):

- Preeclampsia is gestational hypertension accompanied by ≥1 new-onset conditions at or after 20 weeks’ gestation. Eclampsia is defined as seizures in a pregnant woman with preeclampsia with no other identifiable cause. Preeclampsia and eclampsia are classified in the same category (preeclampsia-eclampsia, PE).
- Chronic hypertension (CH) refers to high BP predating the pregnancy or recognized at <20 weeks’ gestation.
- Gestational hypertension (GH) is persistent *de novo* hypertension that develops at or after 20 weeks’ gestation in the absence of features of preeclampsia.
- Chronic hypertension with superimposed preeclampsia (CHSP) is chronic hypertension with development of new proteinuria and/or organ or uteroplacental dysfunction(s).

In this study, the APOs included anemia, diabetes mellitus, preterm birth, low birthweight, hemorrhage disorder, infection, liver disease, stillbirth and neonatal death, maternal near-miss, kidney disease, heart disease, pulmonary disease, embolism, and maternal death. The definitions of the above APOs were consistent with the WHO standards (17, 18).

### 2.3 Ethics approval and consent to participate

The Medical Ethics Committee of Hunan Provincial Maternal and Child Health Care Hospital approved the study (NO: 2024-S155).

The Hunan Provincial Health Commission routinely collected surveillance data, and the government has developed the “Maternal Near Miss Surveillance Working Manual” to collect those data. It is a retrospective study of medical records; all data were fully anonymized before we accessed them. Moreover, we de-identified the patient records before analysis. Since the Government routinely collects these data, the need for informed consent to participate was waived. We confirmed that all operations were following relevant guidelines and regulations.

### 2.4 Data quality control

The Hunan Provincial Health Commission formulated the “Maternal Near Miss Surveillance Working Manual” for surveillance. Data were collected and reported by experienced and trained doctors and nurses. To ensure data consistency and accuracy, all collectors must be trained and qualified before starting work. The Hunan Provincial Health Commission asks the technical guidance departments to conduct comprehensive quality control yearly to reduce surveillance data integrity and information error rates.

### 2.5 Statistical analysis

We calculated the prevalence of HDP and 95% confidence intervals (CI) by the log-binomial method (19). A Pearson’s chi-square test (*χ^2^*) was used to compare differences in the prevalence of APOs across the four HDP subtypes. A *p*-value of <0.05 was considered statistically significant.

Statistical analyses were performed using SPSS 22.0 (IBM Corp., NY, United States).

## 3 Results

### 3.1 Prevalence of HDP

A total of 780,359 pregnant women were included in this study, and 38,397 women with HDP were identified, including 17,782 PEs (46.31%), 15,889 GHs (41.38%), 3,340 CHs (8.70%), and 1,386 CHSPs (3.61%). The prevalence of HDP, PE, GH, CH, and CHSP was 4.92% (95%CI 4.87–4.97), 2.28% (95%CI 2.25–2.31), 2.04% (95%CI 2.00–2.07), 0.43% (95%CI 0.41–0.44), and 0.18% (95%CI 0.17–0.19), respectively (Table 1).

**Table 1:** Prevalence of hypertensive disorders of pregnancy in Hunan Province, China, 2012–2022.

Year	Pregnant women (*n*)	HDP (*n*)	Prevalence (%, 95CI)	Subtypes							
				PE (*n*)	Prevalence (%, 95CI)	GH (*n*)	Prevalence (%, 95CI)	CH (*n*)	Prevalence (%, 95CI)	CHSP (*n*)	Prevalence (%, 95CI)
2012	62,608	1944	3.11 (2.97–3.24)	1,229	1.96 (1.85–2.07)	574	0.92 (0.84–0.99)	81	0.13 (0.10–0.16)	60	0.10 (0.07–0.12)
2013	67,886	2,825	4.16 (4.01–4.31)	1,691	2.49 (2.37–2.61)	933	1.37 (1.29–1.46)	137	0.20 (0.17–0.24)	64	0.09 (0.07–0.12)
2014	75,817	3,053	4.03 (3.88–4.17)	1,674	2.21 (2.10–2.31)	1,131	1.49 (1.40–1.58)	165	0.22 (0.18–0.25)	83	0.11 (0.09–0.13)
2015	79,513	3,127	3.93 (3.79–4.07)	1,629	2.05 (1.95–2.15)	1,223	1.54 (1.45–1.62)	142	0.18 (0.15–0.21)	133	0.17 (0.14–0.20)
2016	71,310	2,950	4.14 (3.99–4.29)	1,387	1.95 (1.84–2.05)	1,202	1.69 (1.59–1.78)	197	0.28 (0.24–0.31)	164	0.23 (0.19–0.27)
2017	78,919	3,518	4.46 (4.31–4.61)	1,673	2.12 (2.02–2.22)	1,437	1.82 (1.73–1.92)	287	0.36 (0.32–0.41)	121	0.15 (0.13–0.18)
2018	76,647	3,943	5.14 (4.98–5.30)	1804	2.35 (2.25–2.46)	1763	2.30 (2.19–2.41)	283	0.37 (0.33–0.41)	93	0.12 (0.10–0.15)
2019	75,033	4,127	5.50 (5.33–5.67)	1,636	2.18 (2.07–2.29)	1984	2.64 (2.53–2.76)	427	0.57 (0.52–0.62)	80	0.11 (0.08–0.13)
2020	68,756	4,120	5.99 (5.81–6.18)	1,695	2.47 (2.35–2.58)	1860	2.71 (2.58–2.83)	427	0.62 (0.56–0.68)	138	0.20 (0.17–0.23)
2021	64,101	4,371	6.82 (6.62–7.02)	1,678	2.62 (2.49–2.74)	1839	2.87 (2.74–3.00)	647	1.01 (0.93–1.09)	207	0.32 (0.28–0.37)
2022	59,769	4,419	7.39 (7.18–7.61)	1,686	2.82 (2.69–2.96)	1943	3.25 (3.11–3.40)	547	0.92 (0.84–0.99)	243	0.41 (0.36–0.46)
Total	780,359	38,397	4.92 (4.87–4.97)	17,782	2.28 (2.25–2.31)	15,889	2.04 (2.00–2.07)	3,340	0.43 (0.41–0.44)	1,386	0.18 (0.17–0.19)

HDP, hypertensive disorders of pregnancy; PE, preeclampsia-eclampsia; GH, gestational hypertension; CH, chronic hypertension; CHSP, chronic hypertension with superimposed preeclampsia; CI, confidence intervals.

### 3.2 APOs across HDP subtypes

The overall prevalence of APOs among pregnant women with HDP was as follows: anemia (30.78%), diabetes mellitus (20.33%), preterm birth (19.53%), low birthweight (18.74%), hemorrhage disorder (9.07%), infection (4.71%), liver disease (2.55%), stillbirth and neonatal death (2.38%), maternal near-miss (1.57%), kidney disease (0.98%), heart disease (0.65%), pulmonary disease (0.11%), embolism (0.03%), and maternal death (0.02%). There were significant differences in the prevalence of all APOs between HDP subtypes (*p* < 0.05), except for pulmonary disease, embolism, and maternal death (*p* > 0.05).

The following characteristics describe the prevalence of APOs in different HDP subtypes: anemia was more common in PE (32.97%) than other subtypes; diabetes mellitus was more common in CH (25.27%) and CHSP (25.11%) than other subtypes; preterm birth was more common in CHSP (37.01%) and PE (27.76%) than other subtypes; low birthweight was more common in CHSP (31.39%) and PE (27.72%) than other subtypes; hemorrhage disorder was more common in PE (9.86%) than other subtypes; infection was more common in PE (5.20%) and CHSP (5.05%) than other subtypes; liver disease was more common in CHSP (4.11%) than other subtypes; stillbirth and neonatal death were more common in CHSP (6.64%) and PE (3.18%) than other subtypes; maternal near-miss was more common in PE (2.47%) and CHSP (2.31%) than other subtypes; kidney disease was more common in CHSP (3.97%) and CH (1.44%) than other subtypes; heart disease was more common in CHSP (1.15%) and CH (0.96%) than other subtypes (Table 2).

**Table 2:** Adverse pregnancy outcomes across hypertensive disorders of pregnancy subtypes.

APOs	HDP (*N* = 38,397)		PE (*N* = 17,782)		GH (*N* = 15,889)		CH (*N* = 3,340)		CHSP (*N* = 1,386)		χ^2^ (*Fisher)	P (*Fisher)
	*n*	%	*n*	%	*n*	%	*n*	%	*n*	%		
Anemia	11,819	30.78	5,862	32.97	4,720	29.71	845	25.30	392	28.28	99.62	<0.01
Diabetes mellitus	7,806	20.33	3,394	19.09	3,220	20.27	844	25.27	348	25.11	86.86	<0.01
Preterm birth	7,498	19.53	4,936	27.76	1,506	9.48	543	16.26	513	37.01	2080.11	<0.01
Low birthweight	7,194	18.74	4,930	27.72	1,460	9.19	369	11.05	435	31.39	2170.16	<0.01
Hemorrhage disorder	3,484	9.07	1754	9.86	1,315	8.28	295	8.83	120	8.66	26.23	<0.01
Abortion-related hemorrhage	96	0.25	26	0.15	19	0.12	43	1.29	8	0.58	100.06*	<0.01*
Ectopic pregnancy	15	0.04	2	0.01	4	0.03	8	0.24	1	0.07	22.80*	<0.01*
Ruptured uterus	19	0.05	11	0.06	6	0.04	0	0.00	2	0.14	4.75*	0.13*
Placenta praevia	608	1.58	293	1.65	221	1.39	58	1.74	36	2.60	13.90	<0.01
Abruptio placentae	545	1.42	368	2.07	118	0.74	32	0.96	27	1.95	113.57	<0.01
Tear of soft birth canal	454	1.18	170	0.96	228	1.43	45	1.35	11	0.79	19.04	<0.01
Uterine atony	1707	4.45	853	4.80	708	4.46	111	3.32	35	2.53	27.11	<0.01
Retained placenta	317	0.83	158	0.89	123	0.77	28	0.84	8	0.58	2.43	0.49
Infection	1807	4.71	925	5.20	656	4.13	156	4.67	70	5.05	21.94	<0.01
Abortion-related infection	42	0.11	8	0.04	15	0.09	13	0.39	6	0.43	32.60*	<0.01*
Puerperal infection	50	0.13	25	0.14	18	0.11	5	0.15	2	0.14	0.93*	0.82*
Abdominal surgical site infection	15	0.04	5	0.03	7	0.04	2	0.06	1	0.07	2.45*	0.37*
Urinary tract infection	53	0.14	28	0.16	17	0.11	7	0.21	1	0.07	3.11*	0.35*
Upper respiratory tract infection	632	1.65	325	1.83	240	1.51	45	1.35	22	1.59	7.30	0.06
Thrombophlebitis	5	0.01	4	0.02	0	0.00	1	0.03	0	0.00	4.90*	0.16*
Liver disease	980	2.55	453	2.55	383	2.41	87	2.60	57	4.11	14.89	<0.01
Stillbirth and neonatal death	913	2.38	565	3.18	169	1.06	87	2.60	92	6.64	276.29	<0.01
Maternal near-miss	604	1.57	439	2.47	108	0.68	25	0.75	32	2.31	193.56	<0.01
Kidney disease	378	0.98	183	1.03	92	0.58	48	1.44	55	3.97	160.77	<0.01
Heart disease	250	0.65	122	0.69	80	0.50	32	0.96	16	1.15	15.98	<0.01
Pulmonary disease	44	0.11	25	0.14	14	0.09	2	0.06	3	0.22	4.17*	0.21*
Embolism	11	0.03	9	0.05	2	0.01	0	0.00	0	0.00	4.08*	0.20*
Maternal death	9	0.02	7	0.04	2	0.01	0	0.00	0	0.00	2.45*	0.46*

APO, adverse pregnancy outcome; HDP, hypertensive disorders of pregnancy; PE, preeclampsia-eclampsia; GH, gestational hypertension; CH, chronic hypertension; CHSP, chronic hypertension with superimposed preeclampsia. * When conducting a chi-square test, if any expected frequency is less than 1, or if the expected frequency in more than 20% of the cells is less than 5, we use Fisher’s Exact Test. In the Surveillance Working Manual, some APOs are subdivided into APO subtypes. For example, hemorrhage disorders include various conditions such as abortion-related hemorrhage, ectopic pregnancy, ruptured uterus, placenta praevia, abruptio placentae, tear of soft birth canal, uterine atony, and retained placenta.

## 4 Discussion

Overall, we found that certain APOs were more common in specific HDP subtypes than others. to the best of our knowledge, there relatively fewer studies have compared APOs between HDP subtypes, including China. It may significantly contribute to the field. There are several important findings in this study.

First, most APOs were common in both CHSP and PE than other subtypes, including preterm birth, low birthweight, infection, stillbirth and neonatal death, and maternal near-miss; several APOs were common in CHSP but not PE, including diabetes mellitus, liver disease, kidney disease, and heart disease; and several APOs were common in PE but not CHSP, including anemia and hemorrhage disorder. Previous studies have shown that preeclampsia and eclampsia were more severe than other subtypes of HDP (14, 20, 21). We believe that the main reason for most APOs being common in both CHSP and PE was the presence of preeclampsia and eclampsia. In addition, we found that APOs caused by CHSP seemed to be more severe than PE. Previous studies have reported similar results (22–24). It suggests that CH may complicate preeclampsia and lead to more severe APOs. The results of some previous studies supported our conclusion. For example, Kametas et al. found that superimposed preeclampsia in women with CH was associated with an increased risk of APOs compared with preeclampsia alone (15). However, there are fewer studies comparing APOs across HDP subtypes and even fewer studies reporting the prevalence of APOs of HDP subtypes, especially in China, which makes it difficult to compare our studies with others.

Second, several APOs were common in CH, including diabetes mellitus, kidney disease and heart disease. It is easy to see that these APOs are common nontransmissible chronic diseases (NTCDs). We believe that there are several reasons for these results. On the one hand, CH may be a risk factor for some NTCDs. For example, Kokubo et al. reported that CH was a risk factor for heart disease (25–29), and kidney disease (27, 30). On the other hand, CH and some NTCDs share similar characteristics and causes, which may lead to indirect associations. For example, Tatsumi et al. reported that hypertension and diabetes mellitus are characterized by different pathophysiologies, but they have much in common. Both are categorized as non-communicable diseases caused by similar unhealthy lifestyles, such as heavy alcohol consumption, physical inactivity, and obesity (31); Faulkner et al. reported that both hypertension and heart disease were associated with obesity (32); Younossi et al. reported that type 2 diabetes mellitus and liver disease were associated with obesity (33); Tirapani et al. reported that NTCDs could be both a cause and a consequence of socioeconomic inequalities, the population with lower income is more exposed to risk factors for NTCDs (34).

Third, there were no significant differences in the prevalence of several APOs across HDP subtypes, including pulmonary disease, embolism, and maternal death. However, previous studies have reported inconsistent results. For example, previous studies showed that preeclampsia or eclampsia were associated with amniotic fluid embolism (35); Battarbee et al. reported that CH and associated cardiovascular disease are among the leading causes of maternal and perinatal morbidity and death in the United States (36). We believe that the main reason for the nonsignificant difference in the prevalence of pulmonary disease, embolism, and maternal death across HDP subtypes was the low prevalence, which resulted in biased results.

## 5 Limitations

Some things could be improved in this study. First, we cannot determine whether there is a causal association between HDP and APOs or whether there were other confounding factors. Second, long-term APOs were not included due to data limitations. Third, most of the mechanisms are still unknown. Future in-depth studies are needed. Fourth, the study population were come from a province in China; although the sample size was large, the study nevertheless had limitations in terms of the restricted ethno-sociodemographic characteristics of the study population.

## 6 Conclusion

Most APOs were more common in both CHSP and PE than other subtypes, including preterm birth, low birthweight, infection, stillbirth and neonatal death, and maternal near-miss; diabetes mellitus, liver disease, kidney disease, and heart disease were common in CHSP but not PE; anemia and hemorrhage disorder were common in PE but not CHSP; diabetes mellitus, kidney disease and heart disease were common in CH. Our finding is useful for clinical counseling and future in-depth studies.

## Data Availability

The original contributions presented in the study are included in the article/supplementary material, further inquiries can be directed to the corresponding authors.
